# Polycaprolactone/Starch/Agar Coatings for Food-Packaging Paper: Statistical Correlation of the Formulations’ Effect on Diffusion, Grease Resistance, and Mechanical Properties

**DOI:** 10.3390/polym15193921

**Published:** 2023-09-28

**Authors:** Emanuela Lo Faro, Angela Bonofiglio, Silvia Barbi, Monia Montorsi, Patrizia Fava

**Affiliations:** 1 Department of Life Sciences, University of Modena and Reggio Emilia, via Amendola 2, 42122 Reggio Emilia, Italy; emanuela.lofaro@unimore.it (E.L.F.); angela.bonofiglio01@gmail.com (A.B.); patrizia.fava@unimore.it (P.F.); 2 Department of Sciences and Methods for Engineering, University of Modena and Reggio Emilia, via Amendola 2, 42122 Reggio Emilia, Italy; monia.montorsi@unimore.it; 3 Interdepartmental Research Center for Industrial Research and Technology Transfer in the Field of Integrated Technologies for Sustainable Research, Efficient Energy Conversion, Energy Efficiency of Buildings, Lighting and Home Automation (EN&TECH), University of Modena and Reggio Emilia, Piazzale Europa 1a, 42124 Reggio Emilia, Italy; 4 Interdepartmental Centre BIOGEST SITEIA, University of Modena and Reggio Emilia, Piazzale Europa 1a, 42124 Reggio Emilia, Italy

**Keywords:** biopolymers, food wrapping, design of experiments

## Abstract

Paper is one of the most promising materials for food packaging and wrapping due to its low environmental impact, but surface treatments are often needed to improve its performance, e.g., the resistance to fats and oils. In this context, this research is focused on the formulation of a new paper bio-coating. Paper was coated with liquids containing poly(hexano-6-lactone) (PCL), glycerol and variable percentages of starch (5–10% *w*/*w* PCL dry weight), agar-agar (0–1.5% *w*/*w* PCL dry weight), and polyethylene glycol (PEG) (5% or 15% *w*/*w* PCL dry weight) to improve coating uniformity and diffusion. A design of experiments approach was implemented to find statistically reliable results in terms of the best coating formulation. Coated paper was characterized through mechanical and physical properties. Results showed that agar content (1.5% *w*/*w* PCL dry weight) has a beneficial effect on increasing the resistance to oil. Furthermore, the best coating composition has been calculated, and it is 10% *w*/*w* PCL dry weight of starch, 1.5% *w*/*w* PCL dry weight of agar, and 15% *w*/*w* PCL dry weight of PEG.

## 1. Introduction

In recent times, there has been a significant research emphasis on sustainability in the field of packaging, specifically on finding new ways to enhance the eco-friendliness of packaging materials [1,2]. One of the most widely studied materials for primary and secondary food packaging is paper, a renewable and biodegradable material mainly composed of cellulose sourced from a diverse range of natural origins [3,4]. The positive aspects related to the use of paper and recycled paper are also considered by “The UN Agenda 2030 for Sustainable Development”, where in Section 12.5 it is stated, as one of the main objectives, “by 2030, substantially reduce the production of waste through prevention, reduction, recycling and reuse” [5]. In addition, CONAI (National Packaging Consortium Italy), with the guidelines of 2022, has released a new classification, decreasing the tax for the annual contribution that the users of paper and cardboard packaging must pay, encouraging their use as packaging.

Nevertheless, base paper, also known as uncoated paper, is not ideal for packaging food items with an extended shelf life due to its inherent limitations. These drawbacks include inadequate resistance to microbial activity, low mechanical strength, and a porous microstructure that hinders the prevention of moisture, oils, and oxygen from permeating through. In order to overcome these drawbacks, various advanced functionalization technologies have been extensively studied and developed. For example, paper is commonly coated with chemicals or laminated with aluminum foil or plastic thin films to improve its barrier effect to water vapor, oxygen, mineral oils, and grease [6,7]. The best-performing papers’ coating, having excellent barrier properties, is obtained with polyethylene, fluorinated compounds, and other petroleum-based polymers. On the other hand, when synthetic polymers are used to coat the paper, it significantly diminishes its biodegradability and recyclability. Consequently, the environmental sustainability of such packaging is adversely impacted by these coatings [8]. Indeed, growing concerns regarding the ecological and human health implications of plastic materials and fluorinated compounds (as well as their derivatives) have prompted a quest for alternative solutions in the pursuit of sustainable materials.

Thus, the challenge is finding new eco-friendly and compostable alternatives with the same barrier behavior of polyethylene and fluorinated compounds. To achieve these ecological objectives, the more recent research on sustainable coating is focusing on the use of degradable components of natural origin, such as, for example, biopolymers, polysaccharides, and other materials of natural origin. Several studies have demonstrated that different biodegradable polymers, such as polylactic acid (PLA), polycaprolactone (PCL) or poly 3-hydroxybutyrate-co-3-hydroxyvalerate (PHBV), starch, chitosan and polysaccharides, can be used as barrier coatings for food-packaging paper [8,9,10]. Among those, PCL has been deeply studied as it is a linear thermoplastic aliphatic polyester, partially crystalline, hydrophobic and biodegradable with good mechanical properties and high extensibility [11,12]. Sundar et al. [13] demonstrated that coatings containing PCL (5–25%) reduce the water vapor transmission rate (WVTR) of coated paper by 25% compared to uncoated ones and increase the oil/grease resistance value, reaching a 12/12 score for the TAPPI test kit. Bota et al. have demonstrated that a 10% PCL coating on paper improves the evolution of the water contact angle over time; in fact, 6 s after the deposition of the water drop on the sample, the angle still has a value greater than 15% with respect to uncoated paper [14].

Another promising candidate to develop biodegradable films and coatings is starch. Starch is characterized by a crystalline structure, presenting itself in the form of granules, and can be obtained from several natural sources, such as corn, wheat, and many others. It is soluble in water, biodegradable, and has many industrial applications, playing the role of gelling agent, stabilizer, etc. [15]. Several studies have been carried out to evaluate the possibility of improving the properties of paper and cardboard with the use of starch, particularly enhancing their barrier properties against edible oils [16,17]. These studies have demonstrated that the use of different starch formulations can lead to significant improvements in oil barrier properties, showing, in some cases, a 100% improvement compared to untreated paper. The studies have also shown that the effectiveness of starch coatings in preventing the passage of fat components can vary depending on the specific composition of the starch. For example, Chi et al. showed that the addition of different potato starch coatings led to varying levels of grease resistance in coated paperboard samples [15].

These findings suggest that starch coatings have the potential to significantly improve the oil barrier properties of paper and cardboard, with the specific composition of the starch playing an important role in determining their effectiveness [18]. However, starch has poor mechanical properties compared to conventional synthetic polymers, which limits its use as a packaging material. In fact, the elongation at break for coating or film with starch used for food packaging is reported in the literature to be around 10–20 MPa, in contrast with 40–70 MPa reached by the family of polyethylene polymers [15,19,20]. One of the most promising solutions to overcome problems such as this is to mix starch with another polymer or add functional reinforcing fillers in order to create a composite film, such as blending starch with PCL and/or agar [21,22,23]. Researchers have explored the use of agar and starch to create binary composite films that are compatible with each other. The combination of these materials has shown promising results as their mixtures demonstrate enhanced properties when compared to their individual counterparts [21]. In fact, Choi et al. [21] have developed a colorimetric pH indicator film using biodegradable materials, such as agar and potato starch. According to Guo et al., agar plays a dominant role in determining the structure and properties of starch/agar composites. The researchers discovered that the optimal properties were achieved at a specific starch/agar ratio. The addition of agar to starch resulted in notable enhancements in both tensile strength and elongation at break. However, once the agar content exceeded 50 wt.%, the improvements became insignificant [16]. Finally, Mahuwala et al. have formulated a cassava starch/agar nanocomposite containing Ag and ZnO using the solution casting method and carried out an analysis of the antimicrobial properties [20]. These studies suggest that starch and agar are promising candidates to comply with the European objectives and standards while also accommodating consumer requests relating to the sustainability and recyclability of food packaging, as coating for paper for food packaging.

In this context, the aim of this work is to carry out a statistic-guided improvement of the physical characteristics of base paper coated with biopolymers and other compostable natural materials, with particular focus on food-wrapping papers (e.g., baking papers, wrapping papers for food delivery) while maintaining the food’s adsorption of grease and water at acceptable levels during transport, storage/shelf-life, and consumption. The base paper samples were coated with water-based solutions containing PCL (5% *w*/*v*) and variable percentages of native starch (5–10% *w*/*w* PCL dry weight) and agar (0–1.5% *w*/*w* PCL dry weight). Then, to improve the uniformity and spreadability of the coating, different percentages of plasticizers were added to all formulations. In this regard, poly (ethylene glycol) (PEG) (5% *w*/*w* PCL dry weight or 15% *w*/*w* PCL dry weight) and glycerol (4% *w*/*w* PCL dry weight) were introduced to the starch–agar/PCL blend to improve coating uniformity and diffusion. As further innovation with respect to consolidated research, a design of experiments approach was implemented to obtain statistically reliable results in terms of the best coating composition, with the lowest possible number of tests due to the high number of formulation variables taken into consideration [24]. Mathematical models were implemented for each measured property of the coating to quantitatively calculate how different percentages of the selected chemicals can affect the coating properties and the overall best coating composition.

## 2. Materials and Methods

### 2.1. Materials

In this work, a calendered bleached paper (Advantage MG White High Gloss, Mondi Group, Addlestone, UK) was used, with technical data shown in Table 1, supplied by Serchio Distribuzione (Roma, Italy). According to the technical sheet, the paper is obtained from a long-fiber sulphate pulp. Poly(hexano-6-lactone) (PCL) was purchased from Sigma Aldrich (Taufkirchen, Germany), having average Mw 80,000 g/mol, water content < 0.5%, and melt flow index (160 °C/5 kg) 2.01–4.03. Ethyl acetate (Ethyl acetate—ACS reagent, purity > 99.5%, Mw: 88.11 g/mol) and glycerol (1,2,3-Propanetriol, Glycerin, purity > 99.5%, Mw: 92.09 g/mol) were purchased from Sigma Aldrich (Taufkirchen, Germany). Potato starch (CAS-No 9005-84-9, analytical grade) was purchased from PanReac AppliChem ITW Reagents (Cinisello Balsamo, Milano, Italy); agar-agar was purchased from OXOID, Thermo Fisher Scientific (Rodano, Milano, Italy); polyethylene glycol (PEG) 200 (analytical grade, Density 1.124–1.126 g/cm^3^, Hydroxyl value: 535–590, Mw~190–210 g/mol; Fluka Analytical, Rodano, Milano, Italy) was used as a plasticizer.

### 2.2. Coating Preparations

In the coating formulation, PCL and glycerol concentration were kept constant, respectively at 5% *w*/*v* and 4% *w*/*w* PCL dry weight, whereas the amounts of agar, starch, and PEG were varied among selected ranges from previous preliminary tests [11]. Taking into account 3 variables in the coating formulations, a full factorial design was implemented having a total of 12 different coating formulations, each one repeated at least three times for replication. In Table 2, the different combinations of the 12 solutions are reported divided in 3 sets (SET 1, SET 2, and SET 3).

**Figure 1:**
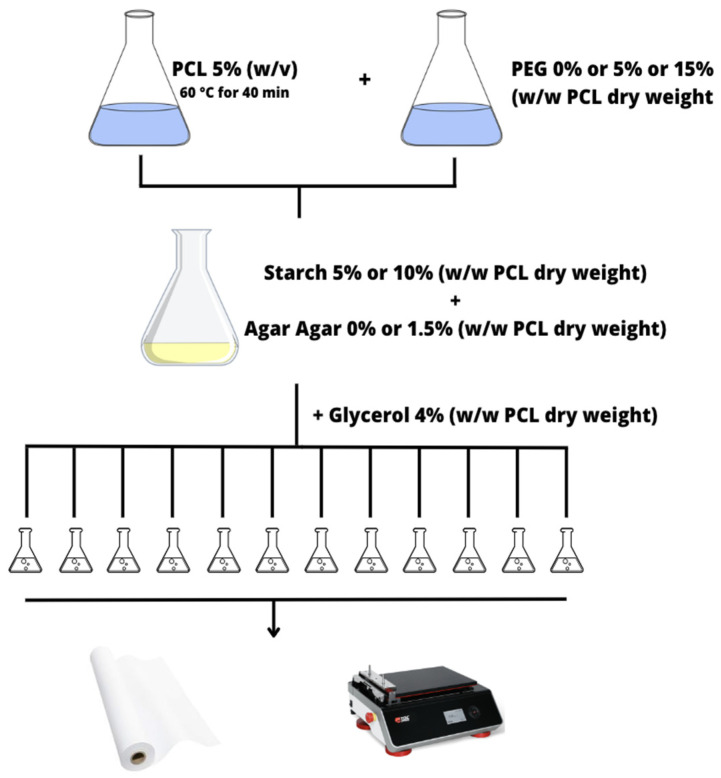
Scheme of the coatings production.

All the solutions were prepared by dissolving 5 g of PCL in 100 mL of previously heated ethyl acetate, under continuous stirring in a water bath at 60 °C for 40 min and with the use of a solvent recondensation system. After the complete cooling of the solution, PEG (5% *w*/*w* or 15% *w*/*w* PCL dry weight) was added whenever required following the experimental plan of the formulations (Table 2). The water solutions containing starch (5% *w*/*w* PCL or 10% *w*/*w* PCL dry weight) and agar-agar (1.5% *w*/*w* PCL dry weight) in their desired concentrations were prepared separately at room temperature. Finally, after the addition of the starch–agar solution to the one containing PCL and PEG to obtain the desirable solution from the design of experiment, 4% (*w*/*w* PCL dry weight) glycerol was added to all the samples. Paper sheets (210 × 297 mm) were coated with the different solutions, employing a bar coating with a Compact AB3650 (TQC Sheen) automatic film applicator, working under a fume hood with the layer-by-layer technique (Figure 1). All paper sheets were coated using the film applicator with a nominal coating thickness of 100 µm and an application speed of 50 mm/s. Once coated with the layer, the paper samples were dried under a fume hood for about 30 min in order to remove the solvent and then dried in an oven at 80 °C for 1 h. The samples were kept for 24 h at room temperature before testing.

### 2.3. Characterizations

#### 2.3.1. Grammage and Thickness Determination

The grammage was calculated by weighing with an analytical balance (accuracy 0.0001 g), with 10 specimens of 1 cm × 1 cm (1 cm^2^) cut randomly from three sheets of each sample of uncoated and coated paper. It is expressed in g/m^2^ (mean value of 10 replicates). A digital micrometer (Syntek, New York, NY, USA) with a sensitivity of 0.001 mm was used to measure the thickness. Three thickness measurements were carried out on both sides and in the central part of 10 rectangular specimens of 150 × 25 mm that were cut from three sheets of uncoated and coated paper (the same specimens were used for the mechanical properties determinations; see Section 2.3.6). A total of 30 measurements were carried out, from which the mean and standard deviation values were calculated.

#### 2.3.2. SEM Analysis

The surface of the paper samples was analyzed by scanning electron microscopy using a Nova NanoSEM 450 (FEI, Hillsboro, OR, USA) equipped with an LVD detector, under low vacuum conditions (80 KPa) and with an acceleration of 10 kV. Images were captured at different magnifications (500–1000×) and tilts (0–40°), thus allowing the visualization of the section of the surface.

#### 2.3.3. Contact Angle Determination

Contact angle (CA) values were measured by means of an OCA 15EC contact angle meter and using OCA 20 (Dataphysics) software by the sessile drop method. For each type of sample, 1 × 10 cm paper strips were positioned on a film holder. CA measurements were taken depositing 3 µL of water or 7 µL of castor oil on the sample surface. For the CA measurement of each type of paper coating samples, 10 replicates were taken, the average of which was considered. The water CA was measured immediately after the drop deposition (t0) and after 15 s (t15) and 30 s (t30) s. Oil CA was measured at different time steps with the same procedure employed for water CA.

#### 2.3.4. Grease Resistance Determination

The grease resistance was tested by using the standard method, namely T 559 pm-02 (or “Kit 12” test) [25]. According to this test, the 12 solutions to be analyzed were prepared by mixing castor oil, heptane, and toluene in adequate portions, thus obtaining solutions that emulate different surface tensions. Solutions with higher numbers are more aggressive, having lower surface energies (i.e., Solution 1 is the less aggressive, while Solution 12 is the most aggressive). A drop of each solution of the kit test was gently dropped onto the surface of each sample and quickly removed with a clean absorbent cloth after 15 s. The tested area was examined and evaluated, giving a specific value to each sample corresponding to the number of the kit test solution that shows the first signs of degradation. Thereafter, the higher the reported value is, higher the resistance of the sample surface to oils is.

#### 2.3.5. Water Vapor Transmission Rate (WVTR)

The WVTR is a measure that indicates the amount of water vapor that can permeate in 24 h through a square meter of a packaging material under defined conditions of temperature and relative humidity [26]. This characteristic is of considerable importance, together with the other diffusion properties, in the development of packaging materials, as it has been demonstrated that increasing the hydrophobic properties of the packaging improves the water vapor barrier properties [27].

The WVTR measurement of the different samples was performed in triplicate according to the ASTM E96 standard method with slight modification [11]. Ten grams of silica gel were put inside a 25 mL glass vials to achieve a 0% internal relative humidity (RH). The samples were glued on the top of the vials, with the coated part inwards to prevent water vapor tangential diffusion, and they were placed in a climate chamber (CH 150—CLIMATEST Climatic Chamber, ARGO LAB) set at 38 °C (+/− 1 °C) and with 90% RH. The vials were weighed 2 times a day for the 5 days of storage. The WVTR value (g 24 h^−1^ m^−2^) was calculated using the following formula:

WVTR = [∆W/(∆t × A)] × 24(1)

where “∆W/∆t” represents the weight gain as a function of time (g·h^−1^), obtained as the slope of the linear regression of the mass gain versus time, and “A” corresponds to the exposed surface of the film (7.85 × 10^−5^ m^2^).

#### 2.3.6. Mechanical Properties

The tensile strength measurements of paper samples were determined by a universal testing machine (BT1- FR1.0TH.140, Ulm, Germany) according to ASTM D882 [28]. For each sample, 20 specimens (dimensions of 150 × 25 mm) were taken: 10 in the machine direction (MD) and 10 in the cross-direction (XD). The dynamometer settings were initial strain 0.1 mm/mm, initial grip separation 125 mm, and speed of grip separation 12.5 mm/min. The collected data were processed by the TESTEXPERT^®^II (V3.31) software. The following mechanical properties have been measured: Young’s modulus (E—MPa), tensile strength (σ—MPa), and elongation at break (ε—%).

### 2.4. Statistical Analysis

A design of experiments (DoE) study was set up to calculate the minimum number of experiments necessary to build up mathematical model, saving time and raw materials. As stated in Section 2.2, three variables were considered, and thereafter a full factorial design was implemented. The other variables that occurred in the process, such as environmental humidity and temperature, were kept the same during all the tests, according to the procedure in Section 2.2. The Design Expert 13.0 (Stat-Ease, Minneapolis, MN, USA) code was used to set up the experimental plan and to perform the statistical analysis of the results. A total of 36 experiments were grouped in the factorial design, consisting also of repetitions for pure error estimation. The central points, were included to calculate the presence of curvature in the mathematical model. All the experimental runs were performed randomly to avoid the presence of systematic errors, following the method reported in Section 2.2.

The data were analyzed firstly by means of PCA (principal component analysis) which made it possible to evaluate the characteristics of the paper samples in a multivariate manner. In particular, the purpose of the PCA analysis in this context was to evaluate which type of paper had overall better performance considering the characteristics evaluated: oil repellency, water repellency, and mechanical strength. Thereafter, a multivariate regression approach through analysis of variance (ANOVA) was set up to mathematically correlate and calculate each formulation effect of the specific evaluated property. The *p*-value (<0.05), related to the F-test, is the statistical parameter necessary to evaluate the significance of the model and each factor. A lack-of-fit test was taken into account to assess a possible significant variation of the experimental points in comparison with their predicted values, R^2^. Adjusted-R^2^ and Pred-R^2^ were calculated to determine the mathematical model fit quality as well as its predictive power. R^2^ is the proportion of the variance in the dependent variables that is predictable from the independent variables, Adjusted-R^2^ is a corrected R^2^ in relation to the number of the performed runs (thereafter aiming to correct any overestimation of the R^2^ due to the increasing number of effects included in the model), and Pred-R^2^ is analogous to R^2^ but associated with predicted values [24].

Finally, a global desirability function was estimated to provide the most desirable combination of factors, considering all the responses analyzed simultaneously. Each response is weighed according to its specific target (Table 3) in terms of objectives and importance, depending on how much each response should match the purpose of the overall study, and then combined using a mean. The desirability function values can be from 0 to 1, where the lowest value (0) represents a completely undesirable combination of independent factors, and, conversely, the highest value (1) indicates a completely desirable (or ideal) combination of them [24].

## 3. Results

### 3.1. SEM Analysis

**Figure 2:**
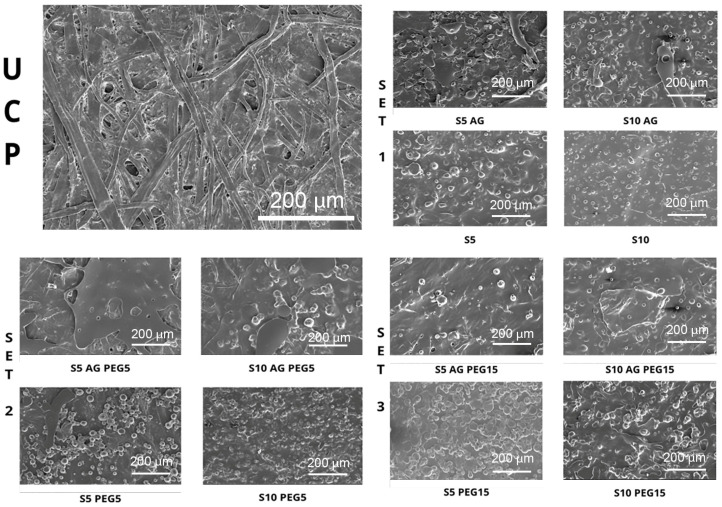
SEM micrographs representative of each investigated sample.

The surface of the uncoated and coated paper samples, analyzed by SEM, is reported in Figure 2. The uncoated paper sample showed the classic open and porous network structure with a non-uniform surface, while in all the coated samples, the typical cellulose fibers and holes of the paper are not visible. For this reason, we can assess that the coating is completely spread in all the samples in a homogeneous way, capable of covering paper fibers and closing paper holes, even if with some differences among them. In fact, the presence of agar (Figure 2) in the coating formulation seems to favor the spreadability of the coating, because the typical hollows of starch are less evident [29,30]. On the contrary, these depressions are bigger and more visible in the samples without agar, especially in the samples which have 10% *w*/*w* PCL dry weight of starch in the formulation of the coating. In addition, comparing the samples with agar of SET 1 (samples without PEG in the coating formulation), with the ones without agar of the same set, the presence of lumps of starch is very clear also in the samples with agar, thus presenting a less homogeneous surface. Already confirmed by our previous studies, PEG strongly influences the spreadability and homogeneity of coatings on cellulosic materials [12]. In fact, as shown in Figure 2, it is possible to see a considerable improvement in the homogeneity of the surface moving from the samples with PEG 5% *w*/*w* PCL dry weight to the ones with 15% *w*/*w* PCL dry weight of PEG. It can therefore be supposed that the PEG/agar combination positively influences the achievement of a more homogeneous and smooth coating surface.

### 3.2. Grammage and Thickness

Grammage and thickness (the last is the thickness of the whole structure, i.e., uncoated paper and paper with coatings) increase in the coated samples with respect to the uncoated samples (see Table 4). Grammage values are almost the same for all the samples due to the amount of coating agents used to create the sample sets and only slight differences can be observed. On the other hand, for the thickness, the presence of agar in the coating solutions lead to significantly higher thickness of the coating layers and this behavior is common for all the three sets. In fact, for the samples coated with solution containing agar the average thickness is around 0.170 mm, while for the samples without agar it is around 0.08 mm. The gelation properties of agar, capable of forming strong gel also at low concentration, may account for an increase of the solution viscosity [16], which probably means that agar does not allow the penetration of the coating solutions into the first layers of the paper structure. These observations are also congruent with the structure of the layers seen in SEM images. It is well known that the addition of a coating generally results in an increase in both thickness and grammage compared to samples without coating, but a more specific calculation must be provided to estimate this increasing effect, both for grammage and thickness. This calculation has been made through PCA and multivariate analysis. As shown in Table 5, the PCA analysis suggests that the only factor that significantly affects thickness is agar. Additionally, samples that include agar in the coating solution have a higher thickness than those without agar; this is evidenced even from the multifactorial ANOVA, with *p* ≤ 0.001. These observations suggest that the specific components and concentrations used in the coating formula can have a direct impact on the resulting thickness of the paper samples, especially the presence of agar. Regarding grammage, there is no statistically significant factor that influences this property.

### 3.3. Oil Contact Angle

**Figure 3:**
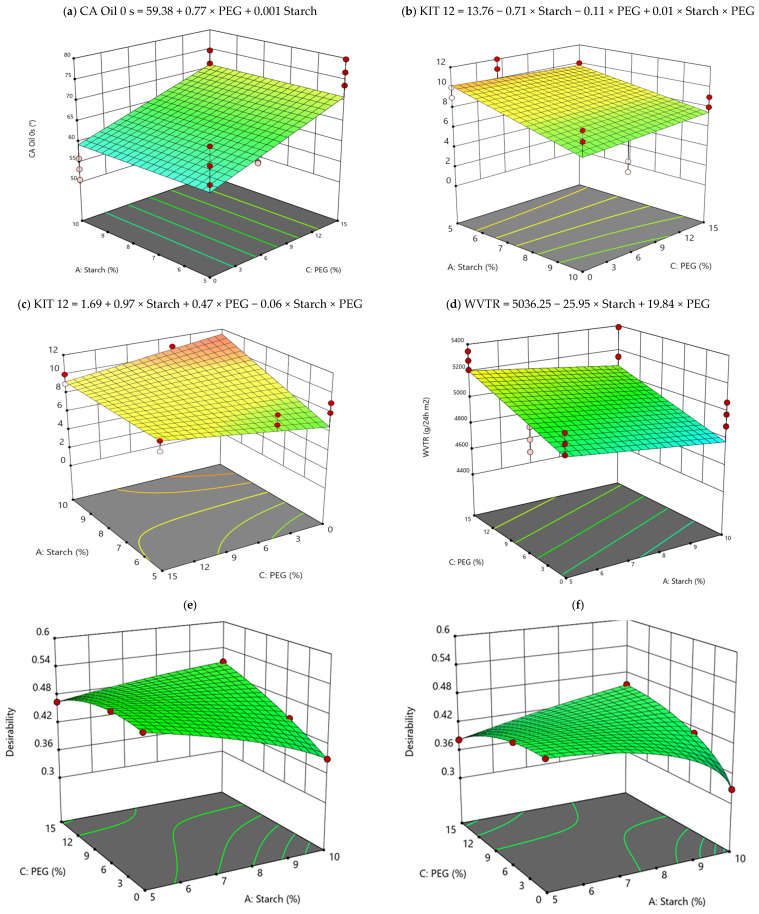
Response surface graph of the most representative mathematical models calculated: (**a**) CA oil 0 s; (**b**) KIT12 with agar; (**c**) KIT12 without agar; (**d**) WVTR with agar; (**e**) Desirability function with agar; (**f**) Desirability function without agar.

In the measurement of the oil CA, castor oil was chosen because it is the basis of the solutions used in the test “Kit-12” (see Section 2.3.4), a reference method to evaluate the repellency of paper against oils and fats. Considering Table 5, the presence of PEG and starch in the formulation of the samples significantly influences the values of the oil CA at 0 s, with *p* ≤ 0.001. Even the interaction between the factors is significant from the statistical point of view but with a restrained influence, as the *p*-value increases (*p* ≤ 0.05). In addition, differences arise among CA measured at different times. As shown in Table S1, after 15 s and 30 s, the agar addition plays the key role, and, thereafter, all the variables considered for coating formulation must be carefully considered in the coating formulation to tailor this fundamental property. In particular, the oil CA after 15 s is around 38° for most of the formulations containing agar and around 32°, for the coating formulation without agar. In strong similarity, after 30 s, both CA decrease, but in the same proportion. In fact, the coatings’ formulation with agar had higher CA, equal to 35°, in contrast with the CA measured in the formulation without agar, that is, 29°. In Table S1 of the Supplementary Materials, the data collected for each sample have been reported. Considering Table S1, in SET 2, sample S5 PEG5 shows the highest CA value at time zero, instead sample S10 AG PEG5 has the highest value at 15–30 s after the drop deposition. As regards the other samples of SET 2, they all have a discontinuous but, at the same time, a rather high value of CA. Samples of SET 1 instead show fairly constant values over time, in particular sample S5, which contains only 5% w/PCL of the starch component. SET 3 samples measured the highest values of all the other samples analyzed in this experiment; in fact, the values recorded at instant 0 s fell within a range between 70° and 77°. In particular, sample S5 PEG15 recorded the highest angle values at time 0. It is important to underline that the oil CA values for all the samples (control paper included) are below 90°, indicating a relative low oleophobicity (conversely a high wettability of the surface), and only the samples of SET 3 seem to show an improvement of the oil CA if compared with the uncoated paper, demonstrating the positive effect of the starch addition to the experimental formulations. In Figure 3a, the response surface graph and the mathematical equation of the model have been reported, estimating quantitatively the effect of PEG and starch on the coating oil CA.

### 3.4. Grease Resistance

The grease resistance of the experimental samples was determined by means of the TAPPI test method procedure named “Kit test 12”, one of the widely used methods to evaluate the performance of paper coatings [31,32]. In this test, 12 different grease solutions (with a surface tension ranging from 0.038 mN/m (less aggressive solution 1, pure castor oil) to 0.022 mN/m (more aggressive solution 12; a mixture of toluene/heptane: 55/45)) are dropped on the coatings, observing traces of staining on the paper. When a material is rated at kit number of 8 and higher it is considered as resistant to grease penetration [10,33]. The data reported in Table 4 show that the uncoated paper has been rated very close to 0 (no resistance against oil and grease), while all the coated samples are characterized by values ranging from 8.33 ± 0.66 to 11.33 ± 0.66. The best performance, in terms of highest resistance to grease (above 10) is recorded for samples containing 5% *w*/*w* PCL dry weight of starch when agar is present (Figure 3b), whereas the agar/starch combination could have a slight negative impact on the oil resistance of coated paper samples when agar is absent from the formulation (Figure 3c). Resistance to oil, which further improves in samples that have only the starch component in the formulation, also tends to increase as the percentage of starch increases (Figure 3c). Finally, it is interesting to note that oil CA measured and results obtained from KIT 12 cannot be correlated to each other, according to their definition. In fact, for example, sample PEG5 S10 AG has a higher CA value at 15 s than the other samples (around 45°) but a very low kit-12 value (4). Another case is sample S10, which managed to resist a greater number of kits, 10, but recorded a very low CA value (Figure S1). Among them, sample S5 PEG15 has high values both for the Kit 12 test and for CA measurement, and this suggests that the mere presence of starch improves the repellency to oil-based substances and, especially, coatings that do not have agar in their formulation have the best fat repellency.

Regarding oil resistance, together with CA, Kit 12 results should also be considered, and from this analysis we can confidently say that layering a coating on uncoated paper significantly improves its oil repellency performance. In fact, according to Table 5, all the variables taken in consideration in this study influence this property in single or in interaction. For example, the combination of starch and agar or even only the presence of starch have a strong effect on the performance of the paper when compared to the uncoated one (*p* ≤ 0.001).

### 3.5. Behavior of Samples with Water

To complete the samples’ characterization, although it was not the focus of this study, the behavior against water of the coated paper samples was also investigated. In this regard, the WVTR value of all samples was measured, and water CA analysis was performed.

#### 3.5.1. WVTR

WVTR represents a measure of the interactions that the water vapor establishes with the cellulosic fibers of paper, from which low values of WVTR mean the obtainment of cellulosic materials less compatible with water and therefore less susceptible to humidity degradation. In Table 4, measured WVTR values for all the samples are shown. Comparing them with the control group (uncoated paper), it is clear that the lowest values were recorded by the formulations of SET2, in which 5% *w*/*w* PCL dry weight of PEG was included. The explanation for this behavior can be found precisely in the presence of PEG, which at that concentration could have favored the homogeneous dispersion of the coating on the paper surface, also promoting the penetration of the coating into the pores of the paper, thus slowing the passage of water vapor. This result is also confirmed by the SEM images shown in Figure 2. Furthermore, the SET 3 samples showed a higher WVTR value than the SET 2 samples. This phenomenon could be explained by the higher percentage of PEG (15% *w*/*w* PCL dry weight) present in the formulations compared to 5% *w*/*w* PCL dry weight of the SET 2 samples. In fact, as the PEG is hydrophilic at higher concentration, it could increase the permeability to water vapor, favoring the hydration of the substrate [30]. In addition, interaction with the other components must be evaluated. As we can see in Table 5, PEG and agar seem to be the most relevant compounds in formulation from the statistical point of view also considering ANOVA. Then, if we focus our attention on the interaction between PEG and starch (Figure 3d), we can notice how 0% *w*/*w* PCL dry weight PEG, 10% *w*/*w* PCL dry weight starch, and 1.5% *w*/*w* PCL dry weight agar is the best combination for the WVTR performance. The presence of agar is influential on the WVTR, as we also see from the SEM analyses, because it helps with the homogeneous deposition of the coating on the paper. On the contrary, we can see that the worst coating composition is reached for 15% *w*/*w* PCL dry weight PEG combined with agar (1.5% *w*/*w* PCL dry weight), and this aspect depends on the hydrophilic nature of the PEG.

#### 3.5.2. Water Contact Angle

Regarding water CA, all the experimental samples have shown a hydrophilic behavior as all the measured angles were below 90° (Table S2 of the Supplementary Materials). Among the various samples, the ones in SET 2, composed of samples containing PEG at 5% *w*/*w* PCL dry weight, recorded the highest CA values, in particular for samples that have agar in their formulation. In the other sets of samples, it is not possible to find significant differences as in the samples present in SET 2. However, we can state that the presence of PEG and agar, and even more their interaction, significantly influences the water CA; this is also confirmed by the multifactor ANOVA analysis (Table 5), where the factors considered and their interaction are shown.

Particular attention has to be focused on the water CA of the uncoated paper, whose value was around 120°, indicating a relatively high hydrophobicity of the samples. This result may be attributed to the high smoothness of the surface of the calendered paper used, a condition which seems to impact positively on wettability (it decreases). From the results of the water CA, it is evident that the presence of the coatings seems to increase the wettability of the surfaces (i.e., an increase of hydrophilicity). This is probably due to the roughness induced by the coatings themselves.

### 3.6. Mechanical Properties

In general, the mechanical properties of paper were determined in two orthogonal directions: the machine direction (MD) and cross-machine direction (XD). Young’s modulus (E) and tensile strength (σ) are expected to decrease from MD to XD. This is due to the fact that machine made paper has more fibers aligned along MD, therefore making it less deformable in that direction but more resistant [30]. We can see from Table 4 that the collected data, independently of the presence of a coating or not, follows this trend, indicating a specific orientation of the papers’ fiber.

**Figure 4:**
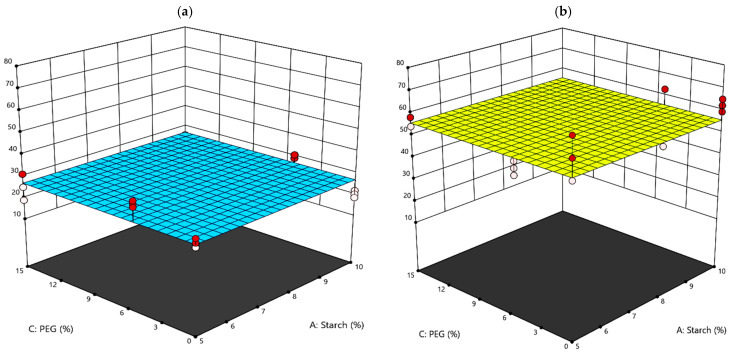
Response surface graphs of the tensile strength XD property: (**a**) AGAR = YES; (**b**) AGAR = NO.

In addition, strong variations are due to the application of a coating and generally a sudden lowering of the recorded value of E and σ can be appreciated with respect to the uncoated paper samples. More in detail, observing the significant values of the multifactorial ANOVA (Table 5), in addition to the direction of the samples, it can be detected that PEG inclusion into the coating formula significantly influences the deformability of the samples. Indeed, PEG acts as a plasticizer, by improving the diffusion of the coating and the deformability of the sample [34]. Thereafter, lower E values are recorded for the samples coated with PEG in their formulation [34]. Indeed, agar plays a significant role in single or in combination with PEG (Table 5). In fact, along the XD, samples containing agar recorded lower σ (27 MPa) with respect to the samples which do not have agar in the coating formulation (56 MPa), regardless of the amount of starch and PEG (Figure 4). Finally, the elongation at break was found to not be significantly affected by the presence of the coatings and therefore was not analyzed further. In summary, the coated paper samples are much more deformable as they reach lower E and σ values, with a consequent decrease in the resistance, which, however, increases with respect to the uncoated paper, in particular for the samples containing only starch in the coating formulation.

## 4. Discussion

In this work, coatings to improve the properties of wrapping paper were formulated by mixing variable percentages of native starch, agar, and PCL. In addition, and as a novelty to the previous literature, to prevent the well-known main problem of the starch-agar/PCL blend, that is the phase separation and the weak interfacial adhesion due to the lack of chemical affinity between these polymers, the addition of an interfacial agent or compatibilizer was considered [23]. The coating formulations explored in this work have been evaluated for their ability to improve or modify some important characteristics of paper, such as wettability (by means the measure of oil and water CA), water vapor transmission rate, and resistance to grease. Concerning wettability, the results obtained measuring the oil and water contact angle demonstrate that the coating solutions do not decrease in a significant way the wettability of the coated paper in comparison with the values of CA measured on the control paper. In fact, regarding oil CA, the average value measured for the control paper is around 66°, in the region of high wettability, according to the “rules of 90° contact angle” [35] and the oil CA of all the three sets of coated samples remained below the critical value of 90°; in particular, the samples of SETs 1 and 2 registered oil CA values very close to those of the control paper, as in a previous work [11]. The samples of SET 3 showed an increase of the oil CA at values greater than 70°, demonstrating the beneficial effect of the simultaneous presence of starch (18) and a high concentration of the hydrophilic plasticizers (PEG) in the formulation, able to improve the homogeneity of the coating [23]. Considering the water CA, uncoated paper showed the higher values (120°) due probably to high smoothness of the calendered surface [35]; in a previous work [11], the same results have been obtained and justified by means the Cassie–Baxter model, which accounts for the contribution of the entrapping of air bubbles into the pores created by the cellulose fibers.

**Figure 5:**
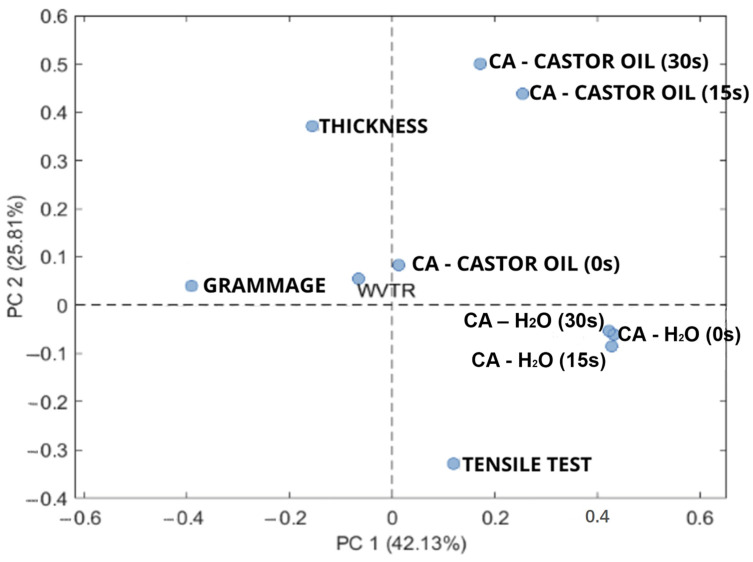
Loading plot of the PCA analysis.

Instead, the relatively low water CA measured on all the coated samples can be explained by the surface roughness induced by the coatings, despite their good spreadability and penetration ability. The WVTR of the coated samples decreased, but not significantly, and only for the SET 1 and SET 2 samples with respect to the average high value determined for the control paper (WVTR 5157 g m^−2^ day^−1^, tropical conditions) despite its high-water CA. There does not seem to be a relationship between water CA and WVTR; in a previous work, for example, it was demonstrated that a commercial fluorinated paper had a very high value of WVTR (2825 g m^−2^ day^−1^) but also a high value of water CA (around 135°), and a polyethylene coated paper had a low WVTR (72 g m^−2^ day^−1^) and a low water CA (around 100°) [11]. If the grease resistance properties are concerned, the results obtained by the Kit Test demonstrated that all the coated samples reached a value higher than 8 despite their oil CA < 90°, confirming that a direct correlation between wettability and oil absorption and diffusion into the paper structure probably does not exist. Taking into account all the previous results, a complete comparison of the obtained data has been reported through PCA and multivariate analysis in which a desirability function has been finally calculated. The optimal dimensionality of the PCA model, i.e., the number of PCs to consider, was defined using the screen plot, a graph that displays the percentage of variance explained by each PC. In this specific study, it was decided to consider two principal components, where PC1 describes 42.13% of the explained variance, while PC2 describes 25.81% of the explained variance. To fully interpret the results of the PCA model, it is necessary to combine the scores graph (Figure S2) with the loading graph (Figure 5). The scores graph allows one to evaluate the relationships between samples, while the loading graph allows for an evaluation of the relationships between output variables. In the score graph (Figure S2), we can see a clear separation of two groups of samples mainly along PC2, namely, the agar-coated group in red (having PC2 positive values), the group without agar in green (with negative PC2 values), and the control samples, represented in blue, clearly separated from the other samples along PC1. It can therefore be stated that PC1 mainly distinguishes between the control samples and the coated paper samples prepared in this study, while PC2 allows for distinguishing between the samples covered with agar and the samples without agar. Comparing the graph of scores with that of loads (Figure 5), we can also see that the samples with agar, positioned at positive values of PC2, show high values of thickness and high levels of CA OIL (0 s, 15 s, and 30 s), as they are variables with positive loading values along PC2, indicating that agar has a more positive influence on these responses, especially in oil resistance. The addition of starch, as also demonstrated by Eslami et al. [36], even at its lowest level (5% *w*/*w* PCL dry weight), is fundamental for this property as it has a relevant influence on the CA measured with oil. The improvement of the grease resistance by the introduction of starch is also demonstrated by the test kit results; all the samples recorded a kit value greater than 8. Samples with any result number higher than 8 are considered grease resistant; similar results were obtained also from Nair et al. [7], and the introduction of starch to obtain the paper coating in their samples led to a marked improvement in oil resistance, with Kit 12 values exceeding 7. In addition, agar presence in combination with PEG has been shown to play a beneficial key role in improving oil resistance and decreasing water vapor transmission rate. Similar results were obtained by Amariei et al. [37], nevertheless, causing a significant detriment to the mechanical properties.

Furthermore, the tensile strength and thickness variables are inversely related to each other, as we can see in the load graph PC1 and PC2. Therefore, at greater coating thickness, lower values of tensile strength are observed. Finally, grammage is inversely related to the water CA value (CA-H_2_O 0 s, 15 s, 30 s), thereafter indicating that some restrictions on the coating formulation must be applied to balance these two responses. For this reason, it is necessary to calculate the best coating formulation to satisfy all the output variables through a desirability function as explained in Section 2.4. In fact, with the desirability function, we can take in consideration the synergic effect among variables and calculate the optimum conditions of work which in this case is the best coating formulation. The results of the calculation of this function are graphically shown in Figure 3, where the graphs are reported for the two conditions: with and without agar, respectively; see Figure 3e,f. It is possible to see the condition with the higher desirability regarding the formulations with agar (Figure 3e) showing desirability over 0.5. More in depth, the calculated best coating formulation is 10% *w*/*w* PCL dry weight starch, 1.5% *w*/*w* PCL dry weight agar, and 15% *w*/*w* PCL dry weight PEG, showing that all the compounds investigated in this study are relevant to produce the more promising coating for packaging paper.

## 5. Conclusions

In the present research, a quantitative calculation of the best performing paper coating for food-wrapping applications has been investigated. Given the growing interest from the food-packaging scientific community in imparting desirable barrier properties to paper [33], agar, PEG and starch, in combination with PCL and glycerol dissolved in water, showed highly functional potential as a polymer matrix for film-coating formation. The application of a design of experiments approach allowed investigating the influence of environment-friendly plasticizer and cross-linking agents on the physical properties of paper coating in a systematic way by clearly identifying composition regions where the formation of a well-balanced coating is promoted and where a synergetic effect can be observed between agar, PEG, and starch. The best coating composition has been calculated, and it is 10% *w*/*w* PCL dry weight of starch, 1.5% *w*/*w* PCL dry weight of agar, and 15% *w*/*w* PCL dry weight of PEG. However, improvements should be made (in terms of new further mixture components) to overcome mechanical property depletion and to achieve a trend comparable to uncoated paper. In addition, further studies regarding degradation mechanisms related to this type of coating should be addressed to evaluate their performance over time and at the end of their life. This study confirms that a well-balanced combination of biopolymers, also from natural origins, could be used to obtain bioplastic coating suitable for the functionalization of paper for food packaging in a circular economy perspective.

## Figures and Tables

**Table 1:** Technical data sheet of uncoated paper.

Properties	Units	Test Method		Values
Basis Weight	g/m^2^	ISO 536		40
Tensile Strength	kN/m	ISO 1924-3	MD CD	4.3 1.8
Tear Strength	mN	ISO 1974	MD CD	340 520
Burst Strength	kPa	ISO 2758		188
Air resistance (Gurley)	S	ISO 5636-5		28
Cobb-60″ MG Side	g/m^2^	ISO 535		21
Gloss	%	TAPPI 480 om-99		25
Brightness	%	ISO 2470		82
Opacity	%	ISO 2471		59
Thickness	μm	ISO 534		55

**Table 2:** Different combination of the 12 coating solutions divided in 3 sets, taking in account the input variables.

		Starch % (*w*/*w* PCL Dry Weight)	Agar % (*w*/*w* PCL Dry Weight)	PEG % (*w*/*w* PCL Dry Weight)
SET 1	S5 AG	5	1.5	0
	S10 AG	10	1.5	0
	S5	5	0	0
	S10	10	0	0
SET 2	PEG5 S5 AG	5	1.5	5
	PEG5 S10 AG	10	1.5	5
	PEG5 S5	5	0	5
	PEG5 S10	10	0	5
SET 3	PEG15 S5 AG	5	1.5	15
	PEG15 S10 AG	10	1.5	15
	PEG15 S5	5	0	15
	PEG15 S10	10	0	15

**Table 3:** Output Variables investigated.

Output Variables	Goal	Importance
WVTR	to minimize	2
Grease Resistance—Kit 12	to maximize	5
Contact angle measurements oil	to maximize	4
Contact angle measurements water	to maximize	2
Thickness	to minimize	3
Grammage	to minimize	3
Mechanical properties	In range	3

**Table 4:** Mean values with standard deviation of the data collected for thickness measurement, grammage measurement, contact angle values with oil and water (^1^ only 0 s time), mechanical property values (Young’s modulus, tensile strength, and elongation at break), Kit Test 12 values, and WVTR values. Results of multifactorial ANOVA are reported as F-values and lowercase letters (“c” > “b” > “a”), respectively. Different letters identify significantly different samples.

SET	Samples	Thickness (mm)	Grammage (g/m^2^)	Oil CA ^1^ (Degrees)	Water CA ^1^ (Degrees)	Kit Test 12	WVTR (g m^−2^ day^−1^) 38 °C 90% RH		Young’s Modulus (MPa)	Tensile Strength (MPa)	Elongation at Break (%)
1	S5AG	0.166 ^abc^ ± 0.010	54.4 ^ab^± 10.9	65.77 ^abcd^ ± 4.85	54.88 ^def^ ± 3.78	9.33 ^bc^ ± 0.6	4943 ^cdb^ ± 74	XD MD	257 ^e^± 320 1790 ^bcde^ ± 1362	12.32 ^ghi^ ± 0.39 27.55 ^efgh^ ± 1.92	3.43 ^a^ ± 0.77 2.91 ^a^ ± 0.29
	S10AG	0.227 ^a^ ± 0.172	61.8 ^a^± 6.9	56.78 ^de^ ± 2.69	49.90 ^f^ ± 5.41	8.33 ^c^ ± 0.6	4987 ^cbd^ ± 87	XD MD	638 ^bcde^ ± 48 1951 ^abcd^ ± 693	9.57 ^i^ ± 0.84 20.10 ^fghi^ ± 1.66	3.41 ^a^ ± 0.13 3.59 ^a^ ± 0.56
	S5	0.073 ^f^ ± 0.004	52.1 ^ab^± 6.6	65.23 ^bcd^ ± 4.18	52.37 ^ef^ ± 5.14	8.67 ^bc^ ± 0.6	4851 ^de^ ± 138	XD MD	247 ^e^ ± 221 511 ^cde^ ± 306	27.83 ^efgh^ ± 1.39 63.52 ^ab^ ± 8.96	3.42 ^a^ ± 0.46 3.30 ^a^ ± 0.65
	S10	0.077 ^f^ ± 0.003	54.8 ^ab^± 7.4	53.22 ^e^ ± 3.10	57.04 ^edf^ ± 3.46	10.33 ^ab^ ± 0.6	4808 ^de^ ± 182	XD MD	458 ^de^ ± 96 677 ^bcde^ ± 348	23.25 ^fghi^ ± 7.22 62.18 ^ab^ ± 2.80	3.58 ^a^ ± 1.30 3.43 ^a^ ± 0.25
2	PEG5 S5 AG	0.140 ^bcdef^ ± 0.017	52.2 ^ab^± 2.6	58.52 ^de^ ± 0.45	80.11 ^b^ ± 7.84	11.33 ^a^ ± 0.6	4907 ^dec^ ± 88	XD MD	477 ^cde^ ± 116 1108 ^bcde^ ± 152	15.06 ^ghi^ ± 1.34 34.88 ^def^ ± 1.41	3.15 ^a^ ± 0.35 3.14 ^a^ ± 0.25
	PEG5 S10 AG	0.151 ^bcde^ ± 0.010	56.7 ^ab^± 1.5	57.49 ^de^ ± 2.77	81.99 ^b^ ± 9.11	4.33 ^d^ ± 0.6	4800 ^de^ ± 51	XD MD	402 ^de^ ± 85 897 ^bcde^ ± 98	12.51 ^ghi^ ± 1.74 29.09 ^efg^ ± 1.65	2.95 ^a^ ± 0.56 2.76 ^a^ ± 0.23
	PEG5 S5	0.101 ^cdef^ ± 0.005	55.0 ^ab^± 4.4	62.22 ^bcde^ ± 0.12	56.42 ^cd^ ± 6.90	8.33 ^c^ ± 0.6	4810 ^de^ ± 140	XD MD	816 ^bcde^ ± 86 1411 ^bcde^ ± 244	23.65 ^fghi^ ± 0.33 51.46 ^bcd^ ± 3.01	3.21 ^a^ ± 0.16 2.96 ^a^ ± 0.25
	PEG5 S10	0.083 ^def^ ± 0.004	55.7 ^ab^± 7.7	59.03 ^cde^ ± 0.42	60.48 ^cd^ ± 3.65	10.33 ^ab^ ± 0.6	4688 ^e^ ± 110	XD MD	598 ^cde^ ±92 1238 ^bcde^ ± 338	23.81 ^fghi^ ± 2.99 49.50 ^bcd^ ± 13.42	3.15 ^a^ ± 0.66 2.79 ^a^ ± 0.93
3	PEG15 S5 AG	0.155 ^abcd^ ± 0.013	49.6 ^bc^± 5.3	70.20 ^abc^ ± 2.61	53.36 ^def^ ± 4.89	9.33 ^bc^ ± 0.6	5279 ^a^ ± 69	XD MD	702 ^bcde^ ± 200 1618^bcde^ ± 108	12.17 ^ghi^ ± 0.46 24.99 ^efghi^ ± 6.14	3.27 ^a^ ± 0.48 2.61 ^a^ ± 0.92
	PEG15 S10 AG	0.182 ^ab^ ± 0.018	56.0 ^ab^± 10.4	70.90 ^ab^ ± 1.13	54.64 ^cdef^ ± 3.81	8.33 ^c^ ± 0.6	5147 ^ab^ ± 116	XD MD	572 ^cde^ ± 144 1106 ^bcde^ ± 186	10.73 ^hi^ ± 0.60 23.95 ^fghi^ ± 1.58	3.35 ^a^ ± 0.34 3.12 ^a^ ± 0.27
	PEG15 S5	0.079 ^ef^ ± 0.004	58.3 ^ab^± 8.1	76.72 ^a^ ± 3.06	57.01 ^cdef^ ± 4.60	8.67 ^bc^ ± 0.6	5152 ^ab^ ± 120	XD MD	1053 ^bcde^ ± 254 2210 ^ab^ ± 224	22.13 ^fghi^ ± 1.80 54.15 ^abc^ ± 4.06	3.09 ^a^ ± 0.59 3.05 ^a^ ± 0.25
	PEG15 S10	0.089 ^def^ ± 0.003	56.1 ^ab^± 8.1	71.17 ^ab^ ± 3.45	61.91 ^c^ ± 4.50	9.33 ^bc^ ± 0.6	5121 ^abc^ ± 176	XD MD	995 ^bcde^ ± 62 1670 ^bcde^ ± 472	22.62 ^fghi^ ± 0.78 50.92 ^bcd^ ± 2.92	3.65 ^a^ ± 0.37 3.28 ^a^ ± 0.14
	UCP	0.067 ^f^ ± 0.001	41.0 ^c^± 1.9	66.37 ^abcd^ ± 3.26	129.46 ^a^ ± 23.05	0.33 ^e^ ± 0.6	5157 ± 152	XD MD	2057 ^abc^ ± 1245 3498 ^a^ ± 1333	41.89 ^cde^ ± 20.16 55.66 ^abc^ ± 3.10	2.92 ^a^ ± 0.32 2.56 ^a^ ± 0.24

**Table 5:** Results of the multifactorial ANOVA and Tukey’s HD test * = *p* ≤ 0.05; ** = *p* ≤ 0.01; *** = *p* ≤ 0.001.

	Agar	Starch	PEG	Agar PEG	PEG Starch	Agar Starch	PEG Agar Starch
Grammage	/	*	/	/	*	/	/
Thickness	***	/	/	*	/	/	/
CA Oil 0s	/	***	***	/	*	/	/
CA Oil 15s	*	/	/	/	/	/	/
CA Oil 30s	*	/	/	/	/	/	/
Grease Resistance	***	***	/	*	***	***	***
CA Water	***	*	***	***	/	*	/
WVTR	***	*	***	/	/	/	/
Tensile Strength	/	/	***	***	*	/	/
Young’s Modulus	***	**	*	***	/	/	/
Elongation	/	/	/	/	/	/	/

## Data Availability

Research data are available in this document and in the related Supplementary Materials submitted.

## Supplementary Materials

The following supporting information can be downloaded at: https://www.mdpi.com/article/10.3390/polym15193921/s1, Table S1: Results of the multifactorial ANOVA and Tukey’s HD test regarding CA oil. Results are reported as F-values and lowercase letters (“c” > “b” > “a”), respectively. Different letters identify significantly different samples. Table S2: Results of the multifactorial ANOVA and Tukey’s HD test regarding CA water. Results are reported as F-values and lowercase letters (“c” > “b” > “a”), respectively. Different letters identify significantly different samples. Figure S1: Numerical results of the Kit 12 test. Figure S2: Scores of the PCA analysis.
