# Supplementary material for: Polycaprolactone/Starch/Agar Coatings for Food-Packaging Paper: Statistical Correlation of the Formulations’ Effect on Diffusion, Grease Resistance, and Mechanical Properties
Source: Polymers (Basel). 2023 Sep 28;15(19):3921. doi: 10.3390/polym15193921 (PMC10574950; doi:10.3390/polym15193921)
Supplement: Supplementary file 1 [file polymers-15-03921-s001.zip › polymers-2614709-supplementary.pdf]

polymers

MDPI

Supplementary Materials

Table S1. Results of the Multifactorial ANOVA and Tukey's HD test regarding CA Oil. Results are reported as F-values and lowercase letters ("c" > "b" > "a"), respectively. Different letters identify significantly different samples.

	SAMPLE NAME	Time (s)		
		**0 s**	**15 s**	**30 s**
SET 1	S5 AG	65.77 ± 4.85^{abcd}	39.6 ± 2.74	37.10 ± 3.73
	S10 AG	56.78 ± 2.68^{de}	29.48 ± 0.36	28.22 ± 0.66
	S5	65.23 ± 4.18^{bed}	37.95 ± 0.91	37.75 ± 1.33
	S1O	53.22 ± 3.10^{e}	26.33 ± 0.62	24.07 ± 2.35
SET 2	S5 AG PEG5	58.52 ± 0.45^{de}	40.88 ± 0.16	37.07 ± 0.33
	S10 AG PEG5	57.49 ± 2.77^{de}	45.28 ± 1.31	39.93 ± 1.17
	S5 PEG5	62.22 ± 0.12^{bcde}	30.78 ± 0.31	27.90 ± 0.52
	S10 PEG5	59.03 ± 0.42^{cde}	29.15 ± 0.02	24.75 ± 0.40
SET 3	S5 AG PEG15	70.20 ± 2.61^{abc}	37.00 +1.75	34.42 ± 1.38
	S1O AG PEG15	70.90 ± 1.13^{ab}	36.25 ± 0.95	34.85 ± 1.95
	S5 PEG15	76.72 ± 3.06^{a}	32.28 ± 2.34	29.88 ± 1.70
	S10 PEG15	71.17 ± 3.45^{ab}	30.6 2± 0.50	27.87 ± 1.16
UCP		66.37 ± 3.26^{abcd}	39.33 ± 5.82	33.38 ± 2.98

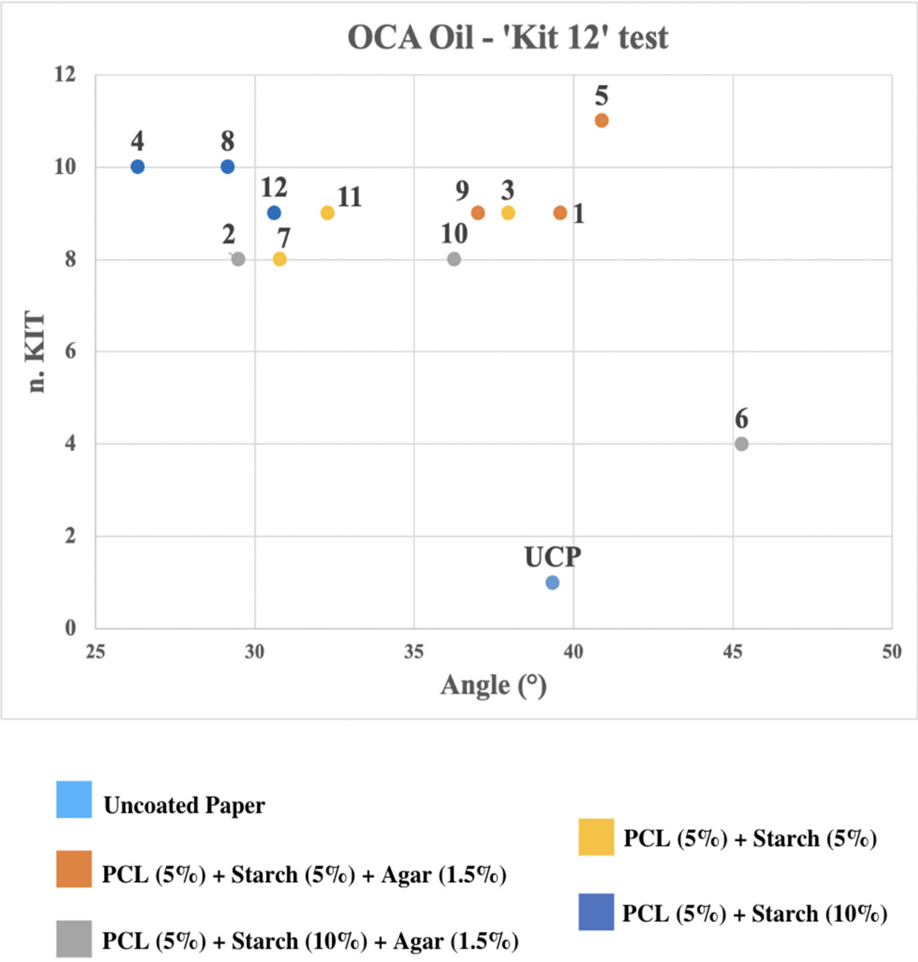

Figure S1. Numerical results of the KIT 12 test.

Table S2. Results of the Multifactorial ANOVA and Tukey's HD test regarding CA Water. Results are reported as F-values and lowercase letters ("c" > "b" > "a"), respectively. Different letters identify significantly different samples.

	SAMPLE NAME	Time (s)		
		**0 s**	**15 s**	**30 s**
SET 1	S5 AG	54.88 ± 3.78^{def}	51.35 ± 6.80	48.50 ± 7.77
	S10 AG	49.90 ± 5.411^{f}	47.74 ± 6.58	46.00 ± 6.67
	S5	52.37 ± 5.14e^{f}	47.91 ± 8.47	44.86 ± 8.69
	S10	57.04 ± 3.46^{cdf}	50.23 ± 6.33	47.39 ± 7.81
SET 2	S5 AG PEG5	30.11 ± 7.48^{b}	78.74 ±7.23	77.74 ± 6.41
	S10 AG PEG5	51.99 ± 9.11^{b}	80.90 ± 8.70	77.52 ± 9.19
	SS PEG5	56.42 ± 6.90^{cde}	52.28 ± 10.33	49.43 ± 8.11
	S10 PEG5	60.48 ± 3.65^{cd}	58.48 ± 5.07	56.50 ± 9.19
SET 3	S5 AG PEG15	53.36 ± 4.89^{def}	47.40 ± 8.34	44.98 ± 8.38
	S10 AG PEG15	54.69 ± 3.81^{cdef}	51.75 ± 7.12	48.95 ± 7.71
	S5 PEG15	57.01 ± 4.60^{cdef}	55.06 ± 5.73	53.65 ± 5.69
	S10 PEG15	61.94 ± 4.50^{c}	61.31 ± 4.83	59.32 ± 5.65
UCP		129.46 ± 23.05^{a}	123.69 ± 3.90	122.05 ± 6.29

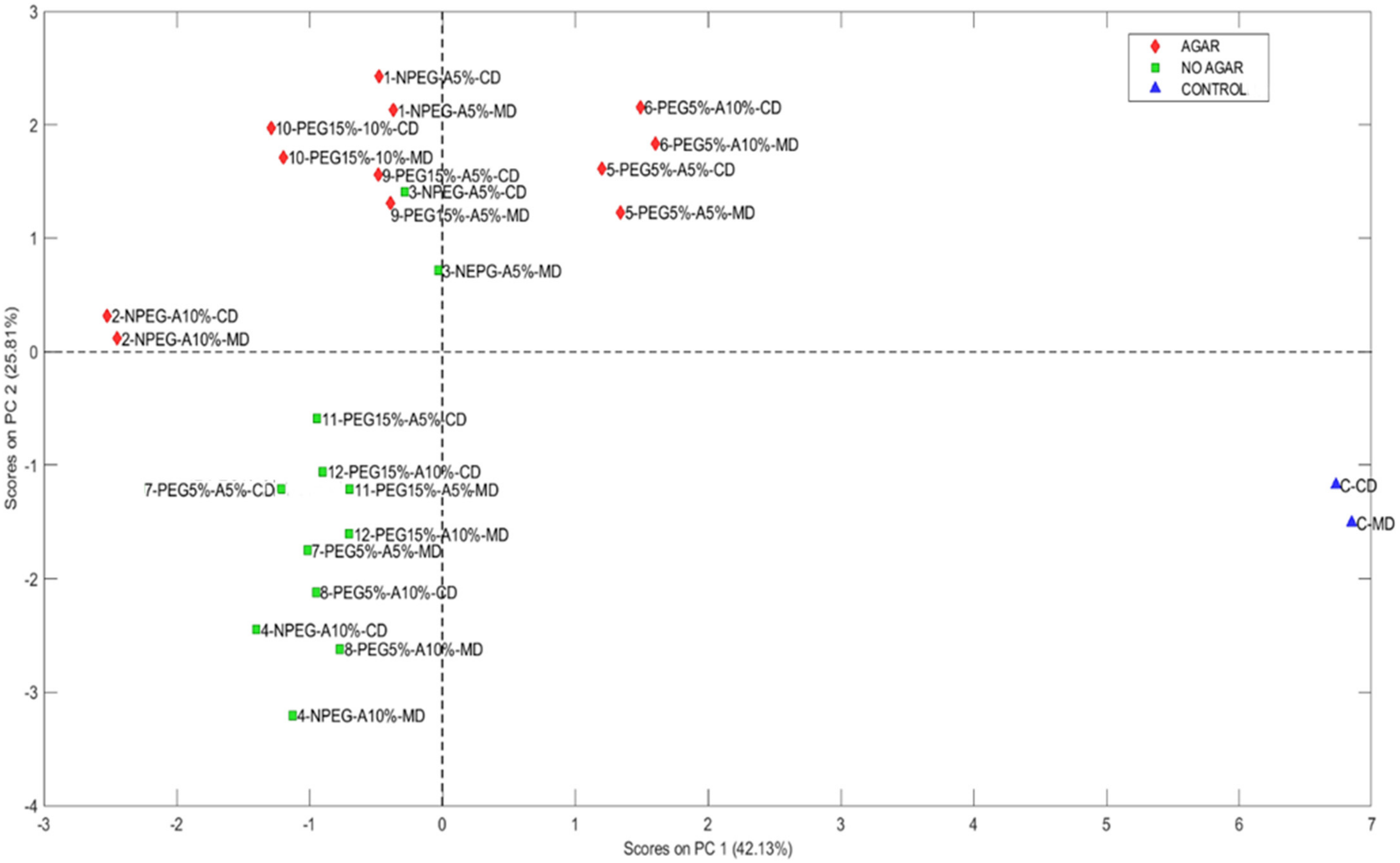

Figure S2. Scores of the PCA analysis.
